# Determinants of meconium-stained amniotic fluid among laboring mother in Ethiopia, systematic review and meta-analysis

**DOI:** 10.3389/fgwh.2024.1393145

**Published:** 2024-07-05

**Authors:** Anteneh Gashaw, Yayeh Adamu, Yohanes Sime, Belete Destaw

**Affiliations:** ^1^ Department of Midwifery, College of Medicine & Health Sciences, Dilla University, Dilla, Ethiopia; ^2^ Department of Anesthesiology, College of Medicine & Health Sciences, Dilla University, Dilla, Ethiopia; ^3^ Department of Psychiatry, College of Medicine and Health Science, Dilla University, Dilla, Ethiopia

**Keywords:** meconium-stained amniotic fluid, labouring mother, systematic review and meta-analysis, determinants, Ethiopia

## Abstract

**Background:**

Meconium-stained amniotic fluid (MSAF) occurs during childbirth when the amniotic fluid carries traces of meconium, the initial stool passed by a newborn. Often signaling fetal distress, MSAF is linked to heightened risks for both the mother and the newborn. In Ethiopia, there is insufficient attention given to this condition. Despite varied study results indicating a considerable range in MSAF occurrences, there is an absence of a comprehensive national overview. Therefore, this systematic review and meta-analysis aim to evaluate the aggregated prevalence of meconium-stained amniotic fluid among laboring mothers and its influencing factors in Ethiopia, providing a consolidated understanding for healthcare strategies and policies.

**Method:**

Following PRISMA guidelines, a systematic review and meta-analysis were executed. Extensive literature searches were conducted on PubMed, Google Scholar, and African Online Journal databases. The pooled prevalence was estimated using a weighted inverse variance random effect model. Heterogeneity among studies was evaluated through Cochrane *Q*-test and *I*^2^ statistics. To assess publication bias, a funnel plot and Egger's test were performed. The identification of factors associated with meconium-stained amniotic fluid among laboring mothers in Ethiopia was conducted using Stata v 18 software.

**Result:**

In total, 63 articles were initially identified, and ultimately, four articles were deemed suitable for inclusion in this review. The combined prevalence of meconium-stained amniotic fluid among laboring mothers in Ethiopia was determined to be 20% (95% CI: 14%–25%). Upon conducting subgroup analysis, it was revealed that the prevalence of meconium-stained amniotic fluid was highest in the Oromia region and lowest in Addis Ababa. Notably, pregnancies complicated by pregnancy-induced hypertension disorder showed a significant association with the presence of meconium-stained amniotic fluid, with an odds ratio of 6.21 (95% CI: 4.04–8.38).

**Conclusion:**

In conclusion, this review emphasizes the common occurrence of meconium-stained amniotic fluid (MSAF). Notably, it identifies a significant association between pregnancy complicated by hypertension and the presence of MSAF. This underscores the need for targeted interventions to reduce MSAF incidence and mitigate associated adverse outcomes in the Ethiopian.

**Systematic Review Registration:**

http://www.library.ucsf.edu/, (CRD42023491725).

## Background

Meconium-stained amniotic fluid (MSAF) is a critical clinical phenomenon encountered during pregnancy and childbirth, often serving as an indicator of fetal distress and potential complications in the neonatal period (1, 2). The presence of meconium in amniotic fluid is associated with an increased risk of adverse outcomes such as perinatal asphyxia, respiratory distress syndrome, and meconium aspiration syndromem (3–6). Understanding the determinants of meconium staining in amniotic fluid is crucial for improving maternal and neonatal health outcomes (7).

Globally, the prevalence of MSAF has been reported to range from 8% to 30% of all deliveries (8–10). However, these figures can vary widely based on the population under study and the specific criteria used to define and diagnose MSAF (11).

In Africa, including Ethiopia, the prevalence of MSAF may be influenced by factors such as maternal age, socio-economic status, and access to healthcare (12). Specific prevalence rates for MSAF in Africa can vary among countries and regions (12, 13).

In an extensive population-based cohort study involving women delivering small for gestational age (SGA) neonates in Israel, significant associations were observed between meconium-stained amniotic fluid (MSAF) and various factors (14). The study identified that gestational age at delivery, maternal age, and nonprogressive first stage of labor independently correlated with the occurrence of MSAF (14). Similarly, a study conducted in Carolina, USA, reported increased risks of MSAF associated with several factors (15). These included advancing gestational age, fetal stress, having fewer than five prenatal care visits, and experiencing labor durations exceeding 15 h (15). These findings underscore the multifaceted nature of determinants contributing to the presence of meconium staining in amniotic fluid, with implications for neonatal outcomes and the dynamics of labor and delivery (14, 15).

Factors influencing MSAF may vary across populations and settings, including maternal age, gestational age, obstetric complications, and socio-economic factors (16). Ethiopia, with its diverse geography, cultural practices, and healthcare infrastructure challenges, provides a rich context for investigating these determinants comprehensively (12). By systematically reviewing and synthesizing the available literature, this study aims to provide a comprehensive understanding of the risk factors associated with MSAF in Ethiopia (17).

In the Ethiopian context, where healthcare disparities and unique socio-economic factors play a role in maternal and neonatal health, there is a need for a comprehensive evaluation of the factors contributing to meconium-stained amniotic fluid (17, 18). This systematic review aims to synthesize existing evidence from diverse studies conducted in Ethiopia to identify and analyze the key determinants associated with the occurrence of meconium staining in amniotic fluid.

Moreover, the inclusion of a meta-analysis component will allow for a quantitative synthesis of relevant data, providing a more robust assessment of the overall impact of specific determinants on the occurrence of meconium staining. The findings of this systematic review and meta-analysis have the potential to inform evidence-based clinical practices, enhance antenatal care strategies, and contribute to the development of targeted interventions aimed at reducing the incidence of meconium-stained amniotic fluid and its associated adverse outcomes in the Ethiopian maternal and neonatal population. Ultimately, this research endeavor seeks to contribute valuable insights that can guide healthcare policies and practices to improve the overall maternal and neonatal health landscape in Ethiopia.

### Research question

What is the prevalence of meconium stained amniotic fluid among labouring mothers in Ethiopia?

What are the determinants of meconium stained amniotic fluid among labouring mothers in Ethiopia?

### Significance of the study

This comprehensive synthesis of evidence is poised to inform healthcare practitioners, policymakers, and researchers, offering a nuanced understanding of the challenges and opportunities in managing laboring mothers at risk of meconium-stained amniotic fluid. By addressing this specific aspect of maternal health, the systematic review aims to contribute to improved antenatal care practices, enhanced labor management, and ultimately better maternal and neonatal outcomes in Ethiopia. And also it has a potential to identify and consolidate crucial information regarding the determinants of meconium-stained amniotic fluid within the unique socio-cultural and healthcare landscape of Ethiopia. Insights derived from this review may shed light on factors such as maternal demographics, obstetric characteristics, and regional variations, providing a foundation for evidence-based interventions and strategies to mitigate the risks associated with meconium staining.

## Method

### Study design and setting

A comprehensive investigation into the determinants of meconium-stained amniotic fluid among laboring mothers in Ethiopia was conducted through a systematic review and meta-analysis. The study adhered to the Preferred Reporting Items for Systematic Review and Meta-Analysis (PRISMA) guidelines, outlined in Supplementary Table S2. PRISMA, designed to enhance transparency and precision in reviews across various fields, including medicine, provides checklists guiding the conduct and reporting of systematic reviews and meta-analyses (19). Ethiopia, located in the Horn of Africa, is classified as a low-income country. As of 2022, the anticipated population is 123.4 million, projected to increase to 133.5 million in 2032 and 171.8 million in 2050 (20). Administratively, Ethiopia is divided into 11 regions and 2 city administrations, with further subdivisions into zones, districts, and kebele, the smallest administrative unit accommodating 2,000–3,500 residents.

### Search strategies and sources of information

We systematically reviewed the PROSPERO database (http://www.library.ucsf.edu/) to identify any existing published or ongoing projects related to our topic, ensuring the avoidance of potential duplication. Our inquiry revealed no ongoing or published articles on this specific topic. Consequently, we registered our systematic review and meta-analysis in the PROSPERO database under the ID number CRD42023491725. To compile relevant literature, we conducted a comprehensive search across international databases, including PubMed, Google Scholar, and African Online Journal. Grey literature was explored using Google. We formulated search terms in adherence to PICO guidelines for online databases, employing Medical Subject Headings (MeSH) and various key terms with Boolean operators such as “AND” and “OR.” The search term used was: “Prevalence” OR “Proportion” AND “Meconium-stained amniotic fluid” AND “Associated Factor” OR “Determinant” AND “Labouring mother” OR “Mother who gave Birth” AND Ethiopia.

### Eligibility criteria

To be incorporated into this systematic review and meta-analysis, studies had to pertain to meconium-stained amniotic fluid in laboring mothers in Ethiopia and be presented in the English language. No restrictions were imposed based on race, and the inclusion period extended until the last search conducted on December 9, 2023. Articles lacking complete abstracts or full texts, along with those not reporting the relevant outcome, were excluded. At each stage of screening, citations lacking abstracts and/or full-text, as well as commentaries, anonymous reports, letters, editorials, reviews, and meta-analyses, were consistently excluded.

### Outcome measurement

This study revolves around two principal outcomes. The primary focus was on determining the prevalence of meconium-stained amniotic fluid among laboring mothers. This was operationally defined as the proportion of laboring mothers experiencing complications related to meconium-stained amniotic fluid. Hence, all the studies included in this analysis aim to identify the presence of meconium-stained amniotic fluid among their respective study participants. The responses was then scrutinized and presented as the prevalence of meconium-stained amniotic fluid in laboring mothers.

The secondary outcome of this study was to investigate the determinants associated with meconium-stained amniotic fluid among laboring mothers.

### Data extraction

All studies retrieved from the designated databases were transferred to Endnote version X 20 software for the purpose of eliminating duplicate studies. Subsequently, all studies were exported to a Microsoft Excel spreadsheet. Independent extraction of essential data was conducted by all authors using a standardized data extraction form, adapted from the Joanna Briggs Institute (JBI) data extraction format.

For the first outcome, which pertains to prevalence, the data extraction format encompassed primary author, year of publication, regions, study area, sample size, and prevalence with 95% confidence intervals. Regarding the second outcome, which involves factors associated with muconium stianed aminotic fluid, data extraction was performed using a 2 by 2 table format.

### Quality assessment

To evaluate the quality of each study incorporated into this systematic review and meta-analysis, the modified Newcastle Ottawa Quality Assessment Scale for cross-sectional studies was employed (21) (refer to Supplementary Table S1). The assessment covered key aspects such as methodological quality, sample selection, sample size, comparability, outcome, and statistical analysis of each study. The responsibility of assessing the quality fell upon two authors (AG, BD), who independently evaluated each study. This evaluation encompassed methodological rigor, sample selection, sample size adequacy, comparability of groups, and the appropriateness of outcome assessment and statistical analysis. In instances of disagreement between the two authors, an additional two authors (YS, YA) were consulted to engage in discussion and resolve any discrepancies.

### Data processing and analysis

The data, formatted in Microsoft Excel, was imported into STATA version 18 for analysis. The pooled prevalence of meconium-stained amniotic fluid among laboring mothers in Ethiopia was estimated using a weighted inverse variance random effect model. To assess heterogeneity among all studies, the Cochran's *Q*-test and I2 statistics were computed. Interpretation of I2 values categorizes 0%–40% as mild heterogeneity, 40%–70% as moderate heterogeneity, and 70%–100% as considerable heterogeneity (22). Funnel plot and Egger's test were utilized to evaluate publication bias, with a *p*-value greater than 0.05 indicating no publication bias. Subgroup analysis was conducted based on the study region. The pooled prevalence of meconium-stained amniotic fluid among laboring mothers, along with 95% confidence intervals, was presented in a forest plot format. To identify determinants of meconium-stained amniotic fluid among laboring mothers, a log odds ratio for each factor was calculate.

## Result

**Figure 1:**
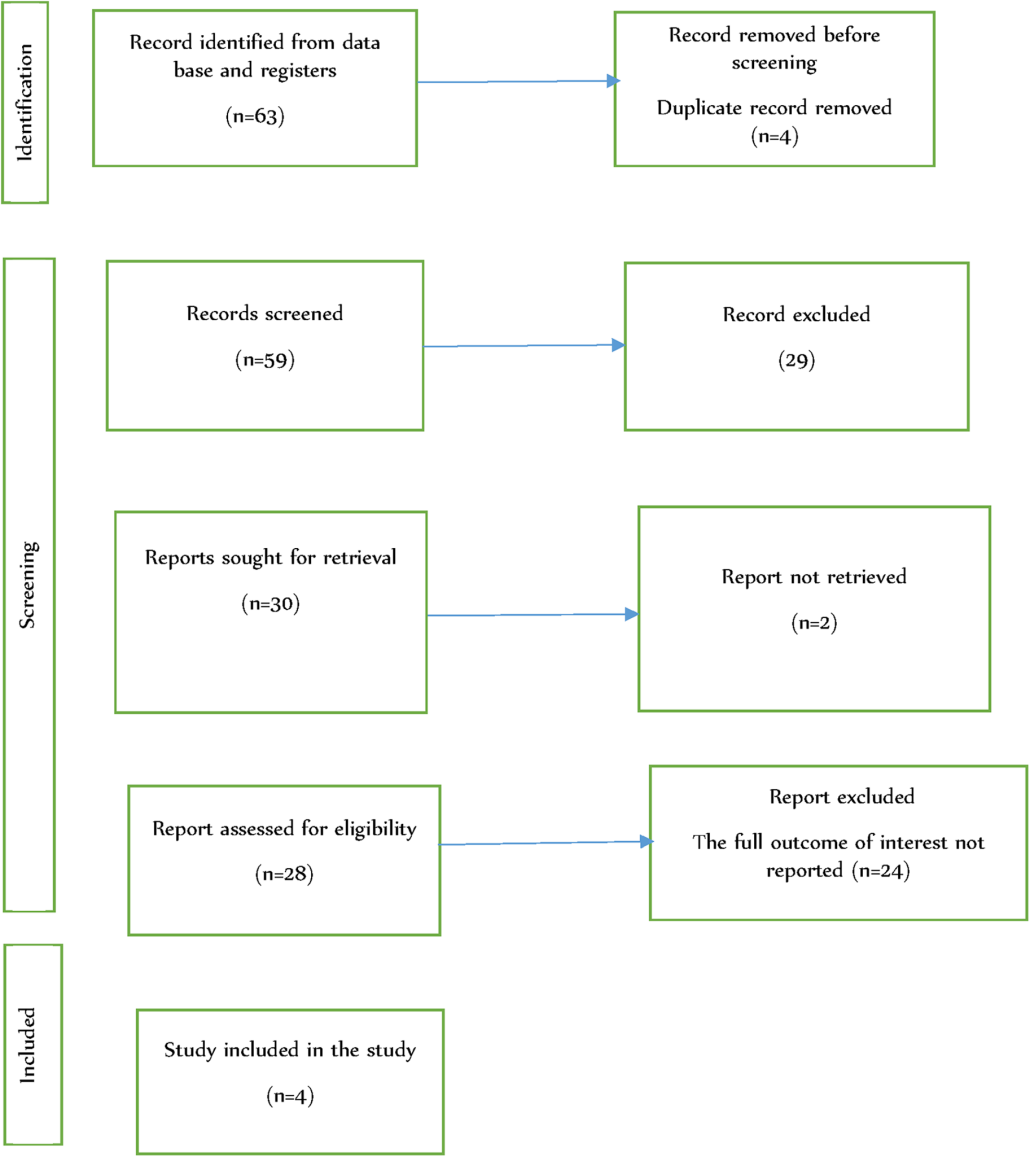
Flow chart of study selection for systematic review and meta-analysis on determinants of meconium stained amniotic fluid among labouring mother in Ethiopia, 2023.

Our search strategy across African Journals Online, Google Scholar, and PubMed databases initially yielded 63 articles. After eliminating duplicates, 59 articles remained. Subsequently, through title and abstract reviews, 10 and 9 articles were excluded, respectively. Following this screening process, 30 full-text papers were thoroughly assessed against inclusion criteria, leading to the exclusion of an additional 26 articles for various reasons. Consequently, 4 papers met the eligibility criteria and were included in the final systematic review and meta-analysis (Figure 1).

Among the studies included in this Systematic Review and Meta-Analysis (SRMA), two were conducted in Amhara, one in Addis Ababa, and one in Oromia. All of these studies were of a cross-sectional nature. The combined participant pool across these studies comprised a total of 1,669 individuals, with the number of participants per study ranging from 248 to 612. The prevalence of meconium-stained amniotic fluid among laboring mothers, as reported by these studies, ranged from 12.1% to 24.6%.

Regarding the quality assessment of the included studies, all of them scored between 8 and 9 on the Newcastle Ottawa Quality Assessment scale, indicating good quality (Table 1).

**Table 1:** Characteristics of included studies in the systematic review and meta-analysis on determinants of meconium stained amniotic fluid among labouring mother in Ethiopia, 2023.

s.no	Author	Year	Region	Study design	Sample	Prevalence	Quality
1	E. Abate	2021	Amhara	Cross sectional	612	24.6	Good
2	D. Addisu,	2018	Amhara	Cross sectional	495	17.8	Good
3	T. Dereje,	2023	Oromia	Cross sectional	314	23.9	Good
4	H. A. Hailemariam	2020	Adiss Ababa	Cross sectional	248	12.1	Good

### Magnitude of meconium stained amniotic fluid among labouring mother in Ethiopia

The combined prevalence of meconium-stained amniotic fluid among laboring mothers in Ethiopia was determined to be 20% (95% CI: 14%–25%). The Cochrane heterogeneity index indicated significant heterogeneity among the different studies (*I*^2^ = 88.45%, *P* < 0.000), surpassing the threshold of 70%. Consequently, to account for and manage this observed heterogeneity, a random-effect model was employed in the analysis. Additionally, to explore potential sources of variation and provide a more nuanced interpretation, subgroup analysis was undertaken.

**Figure 2:**
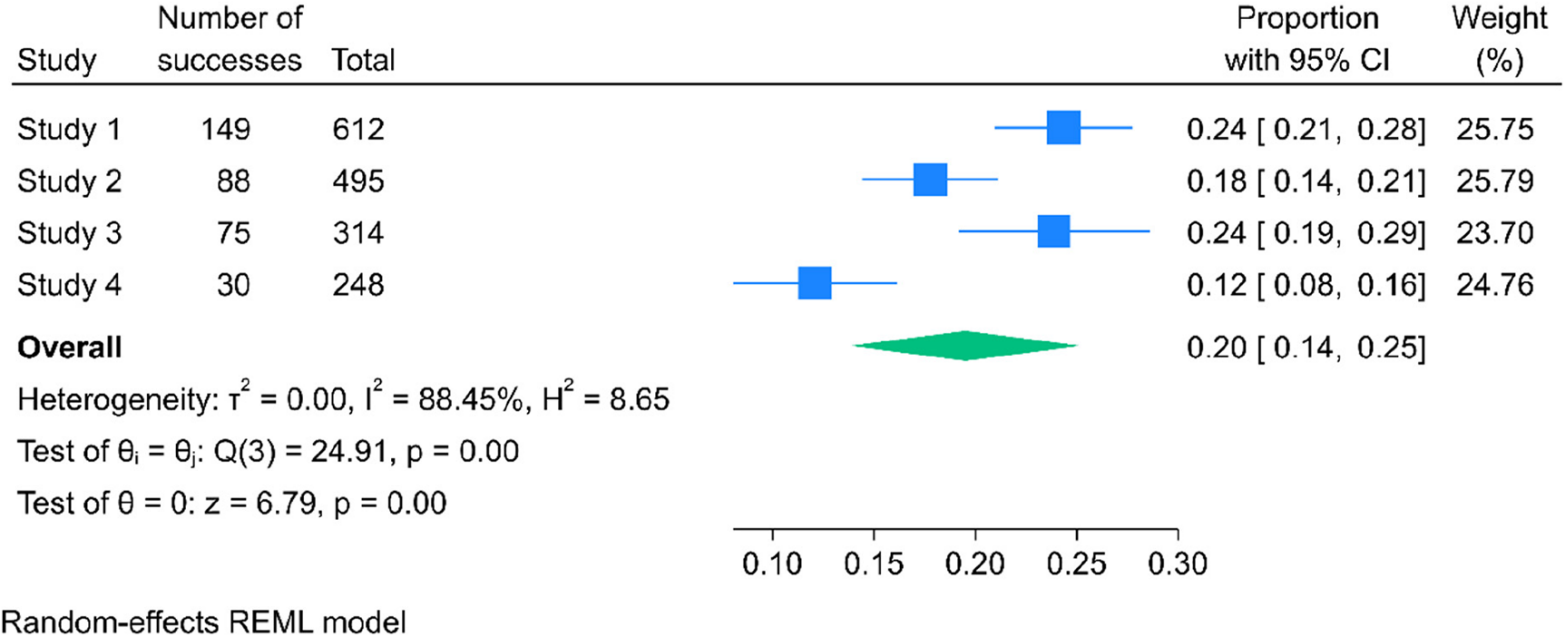
The pooled prevalence of meconium stained amniotic fluid among labouring mother in Ethiopia, 2023.

The results of this analysis are visually presented through a forest plot (Figure 2).

### Publication bias

**Figure 3:**
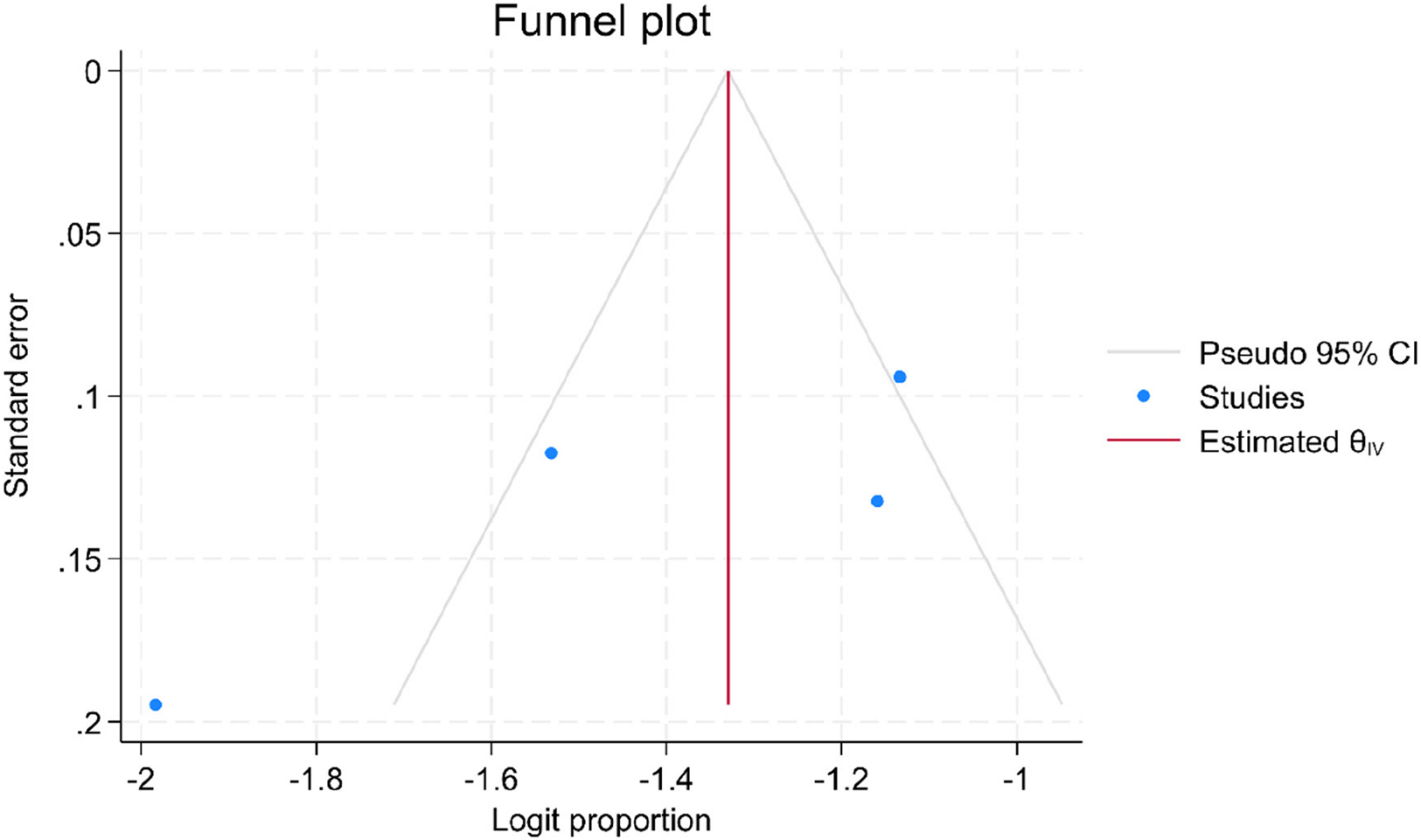
Funnel plot showing the distribution of articles on determinants of meconium stained amniotic fluid among labouring mother in Ethiopia, 2023.

In this systematic review and meta-analysis, the statistical analysis, including funnel plot and Egger's regression test (*P* = 0.27, *p* > 0.05), indicated no evidence of publication bias (Figure 3). However the result of the test might be affected by the small number of included studies and the substantial heterogeneity between included studies.

### Subgroup analysis of determinates of meconium stained amniotic fluid among labouring mother in Ethiopia

**Figure 4:**
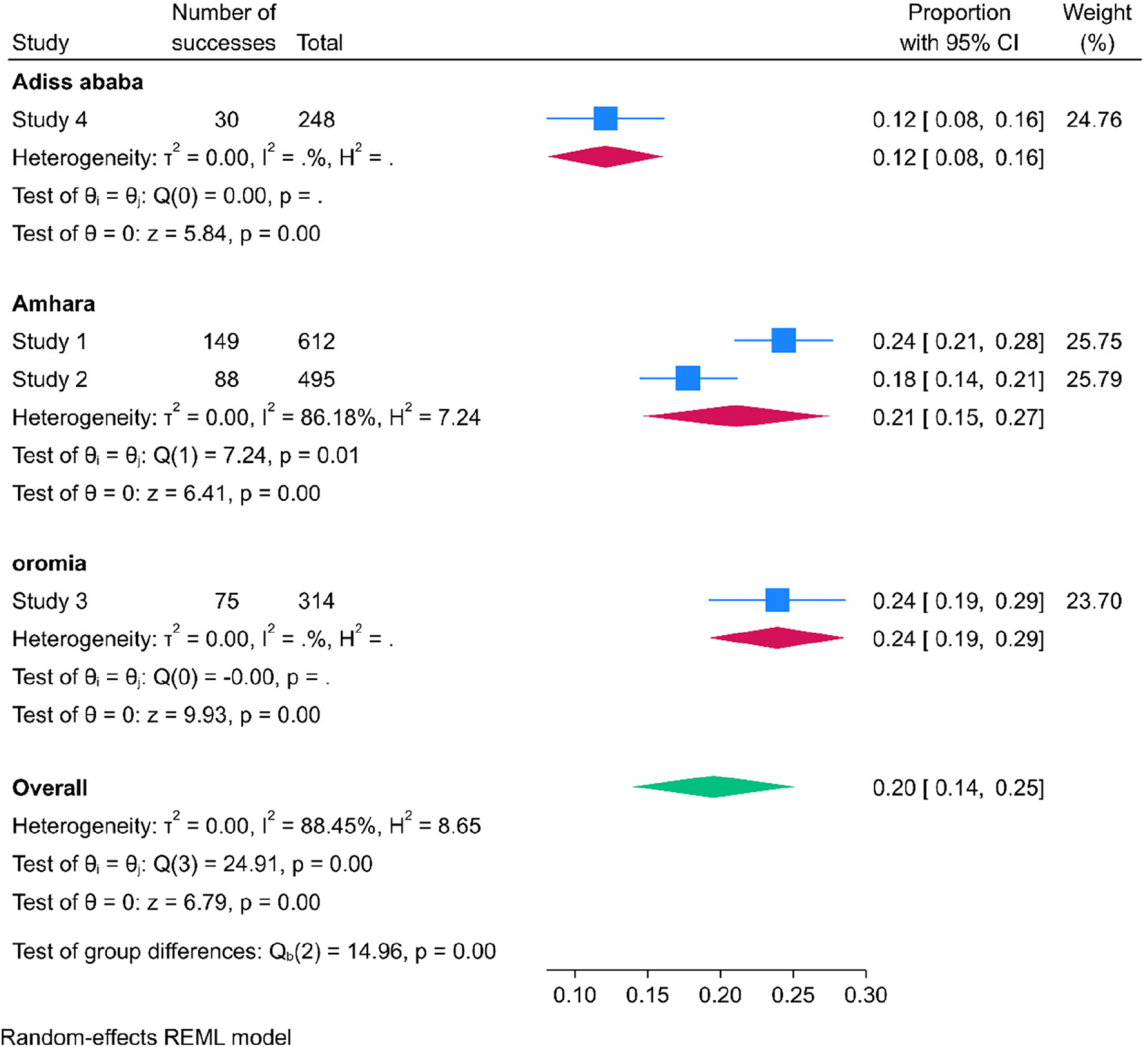
Forest plot showing subgroup analysis of regional determinant of meconium stained amniotic fluid among labouring mother in Ethiopia, 2023.

The finding of subgroup analysis by region showed that the pooled prevalence of meconium stained amniotic fluid among labouring mother was highest in Oromia region [24%; 95% CI: (19%–29%), *I*^2 ^= 0%, *p* = 1] followed by Amhara [21%; 95% CI: (15%–27%), *I*^2^ = 86.18%, *p* = 0.01] then Addis Ababa[12%; 95% CI (8–16), *I*^2^ = 0%, *p* = 1] (Figure 4). In Oromia, another significant regional factor was the prevalence of cesarean deliveries, which stood at 27.7%. Approximately 40% of women in labor experienced complications due to grade three meconium-stained amniotic fluid, and a substantial 72.9% of labors were complicated by prolonged labor lasting over 12 h.

### Sensitivity analysis

**Figure 5:**
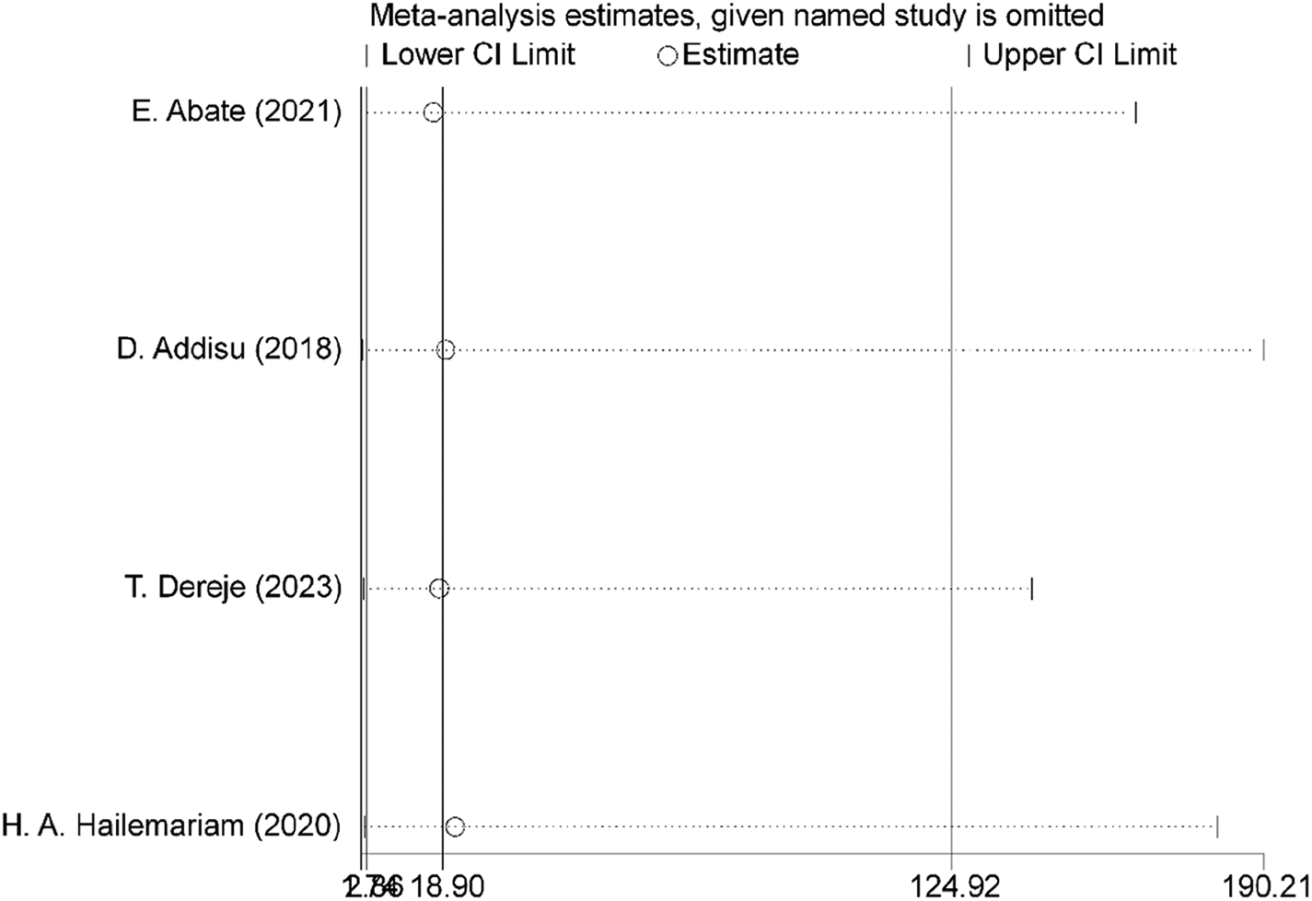
Sensitivity analysis of determinants of meconium stained amniotic fluid among labouring mother in Ethiopia, 2023.

The results from the random-effects model indicated that no single study had a significant influence on the overall pooled prevalence of meconium-stained amniotic fluid among laboring mothers in Ethiopia. This suggests that the combined prevalence was not disproportionately impacted by any individual study, reinforcing the robustness of the overall estimate derived from the collective evidence (Figure 5).

### Determinants of meconium stained aminotic fluid among labouring mother in Ethiopia

In four studies, researchers examined the association between pregnancy induced hypertension and meconium stained amniotic fluid. Of this, all studies (8, 18, 23, 24) showed that pregnancy induced hypertension associated with meconium stained amniotic fluid. The result of this meta-analysis showed that pregnancy complicated with pregnancy induced hypertension was more likely to associate with the prevalence of meconium stained amniotic fluid (OR: 6.21, 95%CI: 4.04–8.38) (Table 2).

**Table 2:** Associated factor with meconium stained amniotic fluid among labouring mother in Ethiopia, 2023.

Variable	Authors	AOR	95%CI	Pooled AOR	95%CI of Pooled AOR
Pregnancy induced hypertension	E. Abate	2.43	1.45, 4.05	6.21	4.04, 8.38
	D. Addisu	3.4	1.26, 9.37		
	T. Dereje	2.48	1.81, 7.54		
	H. A. Hailemariam	4.435	1.624, 12.109		

AOR: is the odds ratio of the respective variable in each primary study.

Pooled AOR: is the point value of odds ratio when we pooled the AOR of primary studies by our analysis.

95%CI of pooled AOR: is the 95%CI of the point value of pooled AOR that is the output of our analysis.

Of the included study that examine the association of post term pregnancy with meconium stained amniotic fluid, three studies (8, 18, 23) showed that post term pregnancy was associated with meconium stained amniotic fluid. And three studies (8, 18, 24) showed that fetal growth restriction was associated with meconium stained amniotic fluid. Furthermore, among the included studies, oligohydraminous, antepartum complication, duration of labour, non-reassuring fetal heart rate pattern, obstructed labour, onset of labour and premature rapture of membrane showed significant association with meconium stained aminotic fluid.

## Discussion

MSAF is often considered as an indicator of fetal distress, Certain factors increase the risk of MSAF, including post-term pregnancy, maternal hypertension, diabetes, and placental insufficiency (25). This systematic review and meta-analysis was conducted to determine the overall prevalence of meconium stained amniotic fluid among labouring mother and identify the determinants of meconium stained amniotic fluid among labouring mother in Ethiopia.

In our study conducted in Ethiopia, we identified an overall prevalence of meconium-stained amniotic fluid (MSAF) at 20%, with a confidence interval of 14%–25%. This discovery aligns closely with findings from related research in Nigeria (20.4%) (26), India which is 16.2% (7) and 18.31% (13). However, it is noteworthy that our study's prevalence is somewhat lower when compared to studies conducted in South Asia, reporting a prevalence of 26% (27) and in India with a prevalence of 29.5% (28). Several factors may contribute to the observed discrepancies. One notable aspect is the consideration of post-term pregnancies in some studies, potentially influencing the reported prevalence rates. Additionally, variations in sample characteristics and methodologies across studies could contribute to differences in the reported prevalence of MSAF. It's crucial to recognize that regional and population-specific factors may play a role in the incidence of meconium staining during labor.

The prevalence of meconium-stained amniotic fluid in our current study, at 20%, is notably higher when compared to a study conducted in India, where the prevalence was reported to be 5.5% (29). This difference in prevalence rates could be attributed to the specific focus on preterm pregnancies in the Indian study. In cases of preterm pregnancies, the development of meconium might occur throughout the course of pregnancy, potentially leading to a lower prevalence compared to studies that encompass pregnancies of varying gestational ages.

In our current study, we observed that pregnancies complicated with pregnancy-induced hypertension were 6.21 times more likely to experience the development of meconium-stained amniotic fluid compared to pregnancies without pregnancy-induced hypertension. This finding is consistent with studies conducted in various regions worldwide (25, 28, 30). The association between pregnancy-induced hypertension and meconium-stained amniotic fluid may be attributed to the fact that pregnancy-induced hypertension poses a direct insult to the fetus. In the presence of any deterioration or insult within the uterus, there is an increased risk of developing meconium-stained amniotic fluid (31). This suggests a potential link between the hypertensive condition and adverse outcomes during pregnancy.

The reason for the differing findings between the current study and previous research, where most studies suggested associations between cesarean section, low APGAR scores (<7 within 5 min), and meconium-stained amniotic fluid (32, 33), could be attributed to random chance, particularly if the sample sizes were small or if the effects being studied were minor. Additionally, variations in the characteristics of the study populations, including demographics, medical backgrounds, or geographical locations, might have influenced the observed relationships.

A study conducted in Ethiopia found that the presence of meconium-stained amniotic fluid (MSAF) can lead to a higher likelihood of cesarean section delivery (32). The research revealed that approximately 15% of laboring mothers with MSAF ended up undergoing cesarean section (32). Additionally, the study observed that around 5% of women with MSAF were diagnosed with elevated blood pressure (34), and the rate of cesarean section increased as the gestational age progressed (32).

While efforts were undertaken to mitigate potential limitations in this study, it is crucial to interpret the results within the context of some acknowledged constraints. Creating direct comparisons became complex due to the lack of similar reviews in other nations, despite our endeavors to synchronize with established meta-analytical outcomes. As a result, we shifted our focus to observational data for discussion, relying on primary studies to grasp the context effectively. Additionally, the limited number of identified studies (only four) through our search strategy, and the absence of studies from specific regions such as Tigray, Dire Dawa, Afar, Gambella, Sidama, Hariri, Somali, and Benishangul-Gumuz, contribute to potential gaps in the comprehensiveness and representativeness of the findings. These limitations emphasize the need for cautious interpretation and underscore the importance of future research efforts to provide a more inclusive understanding of the determinants of meconium-stained amniotic fluid in Ethiopia.

The systematic review conducted in this study focuses exclusively on the Ethiopia region. Consequently, the conclusions drawn from the analysis may have limited generalizability beyond this specific geographical area. While every effort has been made to provide a comprehensive assessment within this context, factors such as cultural variations and healthcare infrastructure peculiar to other regions or countries may influence the applicability of our findings. We acknowledge this limitation and encourage future research endeavors to explore similar topics across diverse geographical settings for a more nuanced understanding.

## Conclusion

In summary, this review underscores that meconium-stained amniotic fluid (MSAF) is a prevalent issue that poses adverse conditions for both the fetus and the newborn. The findings highlight a significant association between pregnancies complicated by pregnancy-induced hypertension and the presence of meconium-stained amniotic fluid. Consequently, these outcomes underscore the necessity for targeted interventions aimed at mitigating the incidence of MSAF and its related adverse outcomes within the Ethiopian maternal and neonatal population.

## Data Availability

The original contributions presented in the study are included in the article/**Supplementary Material**, further inquiries can be directed to the corresponding author.
