# Supplementary material for: Determinants of meconium-stained amniotic fluid among laboring mother in Ethiopia, systematic review and meta-analysis
Source: Front Glob Womens Health. 2024 Jul 5;5:1393145. doi: 10.3389/fgwh.2024.1393145 (PMC11257902; doi:10.3389/fgwh.2024.1393145)
Supplement: Supplementary file 1 [file Datasheet1.pdf]

Supplementary table 1 : Newcastle-Ottawa Quality Assessment Scale for cross sectional studies used in the systematic review and meta-analysis 2022

	Selection				Comparability	Outcome		Total score
Authors	Representativeness (1)	Sample size (1)	Non respondents (1)	Ascertainment of the exposure (risk factor) (2)	The subjects in different outcome groups are comparable, based on the study design or analysis. confounding factors are controlled (1)	Assessment of the outcome (2)	Statistical test (1)	
E. Abate	1	1	1	2	1	2	1	9
D. Addisu	1	1	1	2	1	2	1	9
T. Dereje	1	0	1	2	1	2	1	8
H. A. Hailemariam	1	0	1	2	1	2	1	8

The scoring process was made according to Newcastle-Ottawa Quality Assessment Scale adapted for cross sectional studies

Selection: (Maximum 5 scores)

1) Representativeness of the cases:

a) Truly representative of the HCC patients (consecutive or random sampling of cases). 1 score

b) Somewhat representative of the average in the HCC patients (non-random sampling) . 1 score

c) Selected demographic group of users. 0 score

d) No description of the sampling strategy. 0 score

2) Sample size:

a) Justified and satisfactory (≥ 400 HCC included). 1 score

b) Not justified (<400 HCC patients included). 0 score

3) Non-Response rate

a) The response rate is satisfactory (≥95%). 1 Score

b) The response rate is unsatisfactory (<95%), or no description. 0 score

4) Ascertainment of the screening/surveillance tool:

a) Validated screening/surveillance tool. 2 scores

b) Non-validated screening/surveillance tool, but the tool is available or described. 1 score

c) No description of the measurement tool. 0 score

Comparability: (Maximum 1 scores)

1) The potential confounders were investigated by subgroup analysis or multivariable analysis.

a) The study investigates potential confounders. 1 score

b) The study does not investigate potential confounders. 0 score

Outcome: (Maximum 3 scores)

1) Assessment of the outcome:

a) Independent blind assessment. 2 scores

b) Record linkage. 2 scores

c) Self report. 1 score

d) No description. 0 score

2) Statistical test:

a) The statistical test used to analyze the data is clearly described and appropriate. 1 score

b) The statistical test is not appropriate, not described or incomplete. 0 scor
